# Unveiling Asthma’s Complex Tapestry: Insights from Diverse Perspectives

**DOI:** 10.3390/diagnostics13172798

**Published:** 2023-08-29

**Authors:** Mahesh Padukudru Anand

**Affiliations:** 1 Department of Respiratory Medicine, JSS Academy of Higher Education & Research (JSSAHER), JSS Medical College, Mysore 570015, Karnataka, India; mahesh1971in@gmail.com or pamahesh@jssuni.edu.in; 2 Special Interest Group, Environment and Respiratory Diseases, JSS Academy of Higher Education & Research (JSSAHER), Mysore 570015, Karnataka, India

Asthma, a chronic respiratory disorder affecting millions worldwide, exhibits considerable heterogeneity in its clinical presentation, severity, and response to therapy. As we delve deeper into understanding the underlying genetic and environmental factors that contribute to this complexity, we gain valuable insights into personalized management approaches. In this Special Issue, we embark on a comprehensive journey into the intricate landscape of asthma, unveiling multifaceted facets that have long perplexed researchers and clinicians alike. Through five compelling articles, we illuminate diverse aspects of asthma pathogenesis, biomarkers, and prevalence, collectively shaping a more comprehensive understanding of this complex respiratory disorder.

The original research paper by Sangeetha et al. [1] delves into the genetic association of ADAM33 single-nucleotide polymorphisms (SNPs) with asthma, shedding light on the mechanisms that lead to airway hyperresponsiveness, narrowing, and poor treatment responsiveness. By meticulously genotyping six ADAM33 SNPs, namely rs2280091, rs2787094, rs3918396, rs67044, rs2853209, and rs3918392, the study navigates through the complex interplay between genetics and disease. Of significant clinical importance is the fact that the study not only underscores the association between certain ADAM33 SNPs and asthma susceptibility but also highlights their impact on treatment response. Interestingly, the homozygous minor allele of SNP rs2787094 emerges as a potential genetic marker for predicting poor response to ICS + LABA therapy. This revelation holds immense potential for tailoring treatment strategies to optimize outcomes for individual patients. Furthermore, the identification of SNP rs2853209 as a relevant factor in asthma susceptibility opens new avenues for further investigation. The intriguing link between the minor allele “T” for rs2853209 and moderate disease severity signifies a breakthrough in understanding how genetics contribute to disease progression and severity. The haplotype analysis, which establishes a significant association between specific ADAM33 SNP blocks and asthma, offers a glimpse into the intricate interplay of multiple genetic factors. These findings collectively provide us with a more nuanced understanding of the genetic underpinnings of asthma and open the door for targeted therapeutic interventions. These results should encourage greater exploration of ADAM 33, which is one of the first genes known to be associated with asthma and is probably the most reproducible among all asthma-related genes and has a large potential for transformative shifts in asthma management. The marriage of genetics and medicine is a powerful union that holds promise for delivering tailored interventions, optimizing treatment responses, and alleviating the burden of asthma on patients’ lives.

The second original article by Batra et al. [2] takes us into the realm of statistical methodologies, presenting a refined approach to identifying high asthma admissions and readmissions. By harnessing the power of the Seasonal Hybrid Extreme Studentized Deviate (S-H-ESD) method, this research sets the stage for a more accurate understanding of the disease’s trajectory, thus informing robust interventions. In this article, the authors present the Seasonal Hybrid Extreme Studentized Deviate (S-H-ESD) method as a robust statistical framework and a valuable tool for precisely identifying HAADs and HARDs. This method, known for its high classification accuracy across various contexts, provides a more objective and evidence-based approach than conventional ad hoc definitions. By sidestepping the limitations of ad hoc approaches, the S-H-ESD method emerges as a pragmatic solution for the systematic and accurate classification of HAADs and HARDs. The method’s objectivity and reliability invigorate research endeavors geared toward comprehending the root causes of these admissions and readmissions. In doing so, it lays a foundation for the development of targeted risk mitigation strategies. The adoption of the S-H-ESD method offers a substantial leap forward in accurately identifying and differentiating HAADs and HARDs from background noise. This innovation has the potential to revolutionize the landscape of asthma research, empowering clinicians and researchers alike to refine interventions and policies aimed at reducing the burden of asthma-related hospital admissions.

The first narrative review by Murugesan et al. [3] on one of the important biomarkers relevant to asthma examines the significance of fractional exhaled nitric oxide (FeNO) as a versatile tool in asthma assessment and management. Beyond its role in type 2 inflammation detection, the potential of FeNO to predict responses, adherence, and therapeutic outcomes offers a tantalizing glimpse into tailored treatment strategies. FeNO, a cost-effective and readily available point-of-care biomarker, is renowned for its ability to detect type 2 airway inflammation—an integral component of asthma pathophysiology. Guidelines advocate for its incorporation as an adjunct in the diagnosis of individuals with suspected asthma and the monitoring of airway inflammation. However, the diagnostic utility of FeNO has limitations such as its lower sensitivity, suggesting that it may not be a standalone tool for ruling out asthma. Beyond diagnostics, FeNO encompasses a realm of potential applications. Its capacity to forecast responses to inhaled corticosteroids and predict adherence serves as a beacon of hope for individualized treatment strategies. Moreover, the role of FeNO in guiding the selection of biological therapies is paramount, offering a pathway to optimize therapeutic outcomes. One of the remarkable facets of FeNO is its correlation with disease severity and future asthma exacerbations. Elevated FeNO levels align with reduced lung function and heightened susceptibility to exacerbations, establishing its predictive value. Notably, when integrated with conventional asthma assessment metrics, the predictive accuracy of FeNO is further augmented.

The second narrative review by Sabita Singh et al. [4] introduces a paradigm shift by underscoring the airway epithelium’s dual nature—both a guardian of lung health and a contributor to asthma pathogenesis. This fresh perspective reshapes our comprehension of asthma’s underlying mechanisms and invites innovative interventions aimed at preserving epithelial integrity. The conventional Th2-dominant model has underpinned our understanding of allergic asthma, attributing its features largely to the Th2 immune response. However, emerging insights suggest that a more nuanced perspective—one centered on the airway epithelium’s dynamic role—is needed to address critical gaps in asthma pathogenesis. In this review, the authors unveil an evolving understanding of the airway epithelium’s complex role, from an immune response amplifier to a guardian of lung well-being. This paradigm shift carries implications for designing novel interventions and reimagining asthma management strategies, offering renewed hope for better asthma control and patient outcomes.

Lastly, a systematic review by Ritesh Agarwal et al. [5] delves into the prevalence of aspergillus sensitization and allergic bronchopulmonary aspergillosis in pediatric asthma. By illuminating the epidemiological dimensions, this research emphasizes the need for nuanced community-based studies to comprehend the true prevalence, thus steering more informed clinical decisions. The authors observed a notably high prevalence of AS and ABPA within the realm of pediatric asthma. However, there is a need for robust, community-based studies across diverse ethnicities, employing standardized methodologies.
